# Dissolution improvement of binary solid dispersions of erlotinib prepared by one-step electrospray method

**DOI:** 10.1093/biomethods/bpac001

**Published:** 2022-01-27

**Authors:** Azin Jahangiri, Fakhronnesa Khalilzad, Leila Barghi

**Affiliations:** 1 Department of Pharmaceutics, School of Pharmacy, Urmia University of Medical Sciences, Urmia, Iran; 2 Student Research Committee and School of Pharmacy, Urmia University of Medical Sciences, Urmia, Iran

**Keywords:** Erlotinib, electrospray, solid dispersion, solubility

## Abstract

Erlotinib hydrochloride, a selective tyrosine kinase inhibitor approved for treatment of non-small cell lung cancer firstly. Erlotinib classified as class II drugs in the Biopharmaceutical Classification System (BCS), which characterized by low solubility and high permeability. The aim of this study was to enhance the dissolution rate of this drug. The binary solid dispersions of erlotinib: PVP prepared at different ratios (1:3, 1:5, and 1:8) by electrospray technique. The characterization of formulations performed using differential scanning calorimetery (DSC), Fourier transform infrared spectroscopy (FT-IR) and dissolution rate test. The dissolution results showed that the dissolution rate of erlotinib from binary solid dispersions improved in comparison to pure drug. FTIR spectrum results showed that all peaks of erlotinib functional groups are also observable in the prepared solid dispersions. The FTIR results demonstrated that there was no interaction between drug and polymer. DSC thermograms of the prepared solid dispersions showed no drug-related peak, which is probably related to reduced crystallinity and drug amorphization. Based on the obtained results, it can be concluded that the erlotinib solid dispersion systems displayed improved dissolution rate compared to the pure drug. This will likely lead to increased drug bioavailability.

## Introduction

Poor aqueous solubility of drug candidates is one of the most challenging problems in pharmaceutical research and development [1]. The most important strategy to improve bioavailability of these drugs is their dissolution enhancement.

Erlotinib hydrochloride, N-(3-ethynylphenyl)-6, 7-bis(2-methoxyethoxy)-4-quinazolinamine selectively and reversibly inhibits the epidermal growth factor receptor tyrosine kinase, and therefore prevents the autophosphorylation of tyrosine residues, inhibiting further downstream signaling [2, 3]. Inhibition of tyrosine kinase leads to excessive apoptosis, inhibition of angiogenesis, and finally preventing excessive cell proliferation. Erlotinib is indicated in chemotherapy of metastatic non-small cell lung cancer, pancreatic, head and neck, and ovarian cancer [4]. In the Biopharmaceutical Classification System (BCS) Erlotinib is classified as class II, which is characterized by low solubility and high permeability [5].

The main approaches in order to increase dissolution rate of class II drugs, are particle size reduction, self-emulsification, cyclodextrin complexation, crystal modification, and amorphous solid dispersion [4, 6–8]. Solid dispersion is a family of dosage forms in which the drug component is dispersed in a biologically inert matrix which causes an enhancement in the drug’s oral bioavailability [9]. It has been defined as a product formed by converting a fluid drug-carrier combination to the solid state [10] and although the mechanisms underneath this are poorly understood; it has proven to cause considerable improvements in dissolution rate of the drug [11, 12].

Several methods have been developed for the preparation of solid dispersions including, fusion method, solvent evaporation method, supercritical anti-solvent precipitation process, and electrospraying technique [13].

Electrospraying is recently gaining attention in the production of pharmaceutical nanoparticles and is a method where a high voltage of several kilovolts is applied to a microcapillary nozzle of a spraying system [14]. The highly charged droplets produced in electrospray will travel along the electrical field toward a counter electrode. Electrospray has been successfully utilized for the formation of different drug delivery systems and drugs [15–21].

The purpose of present study was preparation of binary solid dispersion of erlotinib via electrospraying technique using polyvinyl pyrollidone as carrier. Solid dispersions of erlotinib with different drug to polymer ratios (1:3, 1:5, and 1:8) were prepared through electrospraying method and were characterized by Fourier transformed infrared spectroscopy (FTIR) and differential scanning colorimetry (DSC). In vitro drug release test from solid dispersions and corresponding physical mixture was performed.

## Materials and method

### Materials

Erlotinib hydrochloride was synthesized as previously reported [22]. Polyvinyl pyrrolidon (PVP K25) was purchased from Merck chemical company (Germany). Methanol was analytical grade and purchased from Merck chemical company (Germany). Double distilled water was used throughout the study.

### Preparation of solid dispersions

The electrospraying solution was prepared by co-dissolving the polymer and the drug in methanol in order to prepare binary solid dispersion of erlotinib with drug: polymer ratios of 1:3, 1:5, and 1:8 (F_1_, F_2_, and F_3__,_ respectively). Corresponding physical mixtures (P_1_, P_2_, and P_3_, respectively) were also prepared by mixing the polyvinyl pyrrolidone, and the erlotinib by spatula on a flat surface until a uniform mixture achieved. A high-voltage DC power supply was utilized for electrospraying process. The positive electrode of the high-voltage power supply was connected to the needle tip and the grounded electrode was joined to a steel collector. A fixed electrical potential of 15 kV was applied and the collector was electrically ground and placed 15 cm away from the tip of the needle. The prepared polymer solutions were injected with the flow rate of 3 ml/h by a syringe pump. The formed electrosprayed samples were dried overnight at room temperature and then separated from the surface of collector.

### Loading efficiency (%) determination

Loading efficiency (%) was determined by dissolving accurately weighted sample of each formulation in methanol and dilution with hydrochloric acid buffer (pH = 2). Then the amount of drug in each formulation obtained using a UV-visible spectrophotometer at 343 nm (Cecil, England). The loading efficiency expressed in percentage was calculated according to Equation 1 [23, 24].

(1) $$\mathrm{Loading}\;\mathrm{efficiency}\;\left(\%\right)=\frac{\;\mathrm{Actual}\;\mathrm{drug}\;\mathrm{content}}{\;\mathrm{Theoretical}\;\mathrm{drug}\;\mathrm{content}}\times 100$$

### FTIR

FTIR spectra were obtained by an infrared spectrometer (PerklinElmer, USA) using the KBr disk technique in the transmission mode. Data were collected over a spectral region from 400 to 4000/cm with a resolution of 2/cm.

### Thermal analysis

Thermal behavior of erlotinib, prepared solid dispersions and physical mixture as well as, polymer was recorded on a DSC-60 (Shimadzu, Kyoto, Japan). All samples were placed in sealed aluminum and an empty aluminum pan was considered as reference. Thermogram of the samples was obtained at a scanning rate of 10°C/min covering temperature range of 25–300°C.

### *In vitro* drug release

United States Pharmacopoeia dissolution tester apparatus II (Pharmatest, Germany) was applied for evaluating the *in vitro* release of erlotinib from the prepared solid dispersions. The pure drugs, physical mixtures as well as the erlotinib solid dispersions, all equivalent to 25 mg were added into the dissolution medium (900 ml, HCl 0.01 N, pH = 2, under stirring rate of 75 r.p.m., maintained at 37 ± 0.5C). At predetermined time intervals (15, 30, 45, 60, and 75 min), aliquots of 3 ml were withdrawn by using 0.45 μm membrane filter and replaced with fresh medium to maintain constant volume. The sample solutions were analyzed using a UV-visible spectrophotometer at 343 nm (Cecil, England).

## Results and discussion

### Loading efficiency (%)

Loading efficiency (%) expressed in percentage calculated according to Equation 1, are presented in Table 1. Loading efficiency of formulations increased by increasing the polymer content.

**Table 1.** Loading efficiency (%) of prepared formulations

Formulation code	Drug : polymer ratio	Loading efficiency (%)
F_1_	1 : 3	80.68
F_2_	1 : 5	82.26
F_3_	1 : 8	86.12

### FTIR

**Figure 1.**
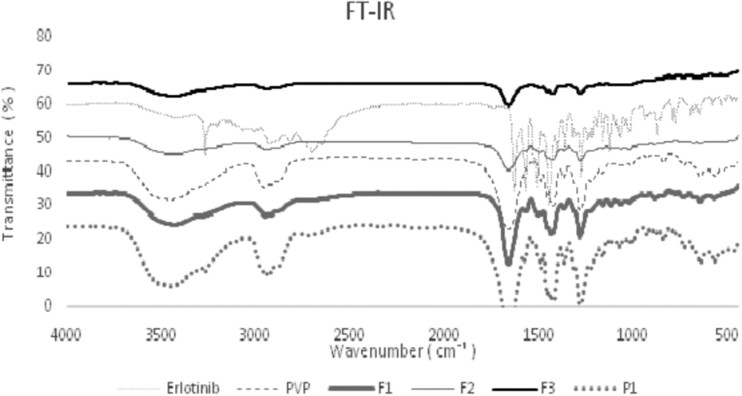
FTIR spectra of erlotinib, PVP, various solid dispersions and physical mixture (P1).

The FTIR spectra of solid dispersions, physical mixture, PVP and erlotinib are demonstrated in Fig. 1. In the erlotinib spectrum, the characteristic peaks at 3273, 2703, 1634, 1450/cm were related to =NH– stretching, ≡C–H stretching, NH bending, Ar–C–N stretching, respectively. Important peaks detected in spectrum of PVP were the 1289, 1668, 2957/cm attributed to C–N stretching, C = O stretching, and C–H stretching, respectively, and the broad band at 3458 was attributed to O–H stretching due to the presence of water. The peaks of the functional groups of erlotinib were detected in the solid dispersion and physical mixture. These results demonstrated that no chemical interaction occurred between the drug and polymer during the solid dispersion preparation process.

### Thermal analysis

**Figure 2.**
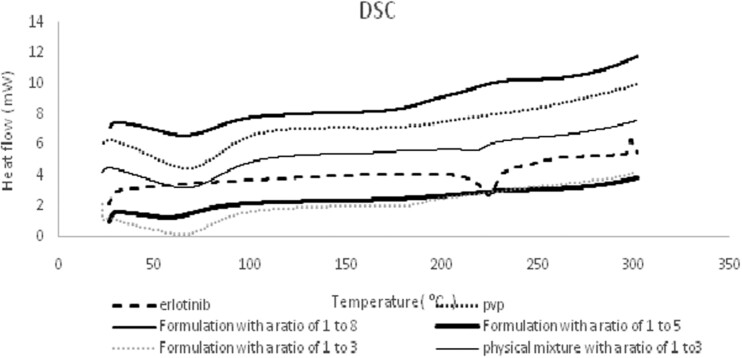
DSC thermograms of erlotinib, PVP, various solid dispersions and physical mixture (P1).

The DSC thermograms of the erlotinib and PVP as well as solid dispersions and physical mixture are represented in Fig. 2. During scanning of the pure erlotinib a single endothermic was observed at 223°C related to the melting point. The DSC thermogram of PVP demonstrated a broad endothermic peak ranging from 45°C to 85°C, due to the loss of water from the hygroscopic PVP. The DSC thermograms of both solid dispersions and physical mixtures displayed a single endotherm of polymer only and the melting endotherm of erlotinib was disappeared owing to the drug amorphization or dilution effect of polymer. Also thermal analysis results demonstrated that no chemical interaction occurred between the drug and polymer during the solid dispersion preparation process.

### *In vitro* drug release

**Figure 3.**
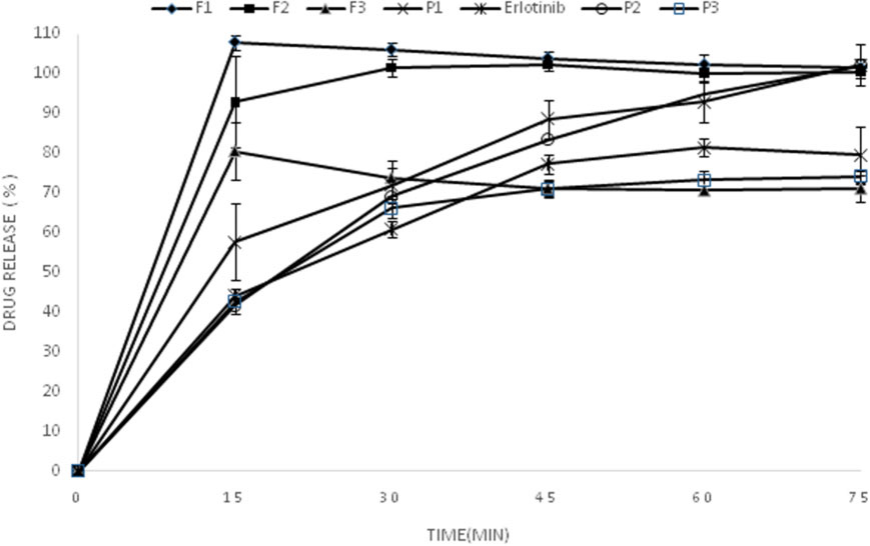
Dissolution profile of erlotinib, various formulations and corresponding physical mixtures.

The *in vitro* drug release profiles of the erlotinib as well as physical mixtures and solid dispersions are represented in Fig. 3. The oral absorption of poorly water-soluble drugs is generally controlled by the dissolution rate in the gastrointestinal tract. Therefore, these drugs exhibit dissolution-dependent bioavailability [25]. Consequently, increase in the dissolution rates of erlotinib as poorly water-soluble drug could possibly lead to an improved oral bioavailability and therapeutic efficacy. The pure erlotinib showed 44% and 80% drug release after 15 and 60 min, respectively, in HCl 0.01 N (pH = 2). Aqueous solubility of erlotinib hydrochloride is pH dependent and maximum solubility occurs at buffer with pH of 2 because of protonation of secondary amine. Binary solid dispersion of erlotinib prepared via electrospray method showed an increase in dissolution rate and drug release compared to the pure drug and physical mixtures, so that the amounts of released erlotinib during the first 15 min from the F_1_ and P_1_ were found to be 100% and 58%, respectively. The amount of released erlotinib during the first 15 min from the F_2_ and P_2_ was 93% and 42%, respectively. Finally, 80% and 42% of erlotinib from the F_3_ and P_3_ was dissolved in 15 min. Therefore, the use of the electrospray as one-step method is a promising technique to enhance the dissolution rate of erlotinib.

## Conclusion

In conclusion, electrospraying, as one-step method, was utilized to prepare binary solid dispersions of erlotinib. The DSC thermograms of both solid dispersions and physical mixture displayed a single endotherm of polymer only and the melting endotherm of erlotinib was disappeared owing to the drug amorphization or dilution effect of polymer. The FTIR results demonstrated that no chemical interaction occurred between the drug and polymer during the solid dispersion preparation process. Solid dispersions of erlotinib prepared via electrospray method showed an increase in dissolution rate and drug release compared to the pure drug and physical mixtures. As the oral absorption of poorly water-soluble drugs is generally controlled by the dissolution rate in the gastrointestinal tract, consequently, increase in the dissolution rates of electrosprayed erlotinib could possibly lead to an improved oral bioavailability and therapeutic efficacy.

## Conflict of interest

The authors declare no conflicts of interest.
